# Repair of critical-sized bone defects in rabbit femurs using graphitic carbon nitride (g-C_3_N_4_) and graphene oxide (GO) nanomaterials

**DOI:** 10.1038/s41598-023-32487-7

**Published:** 2023-04-03

**Authors:** Ahmed Abdelrahiem Sadek, Mahmoud Abd-Elkareem, Hani Nasser Abdelhamid, Samia Moustafa, Kamal Hussein

**Affiliations:** 1 Department of Surgery, Anesthesiology and Radiology, Faculty of Veterinary Medicine, Assiut University, Assiut, 71526 Egypt; 2 Department of Cell and Tissues, Faculty of Veterinary Medicine, Assiut University, Assiut, Egypt; 3 Advanced Multifunctional Materials Laboratory, Department of Chemistry, Faculty of Science, Assiut University, Assiut, Egypt; 4 Proteomics Laboratory for Clinical Research and Materials Science, Department of Chemistry, Faculty of Science, Assiut University, Assiut, Egypt; 5 Nanotechnology Research Centre (NTRC), The British University in Egypt (BUE), Suez Desert Road, El-Sherouk City, 11837 Cairo Egypt

**Keywords:** Medical research, Experimental models of disease, Biomedical materials, Biomineralization, Implants, Biomedical materials, Biomineralization, Implants, Nanobiotechnology

## Abstract

Various biomaterials have been evaluated to enhance bone formation in critical-sized bone defects; however, the ideal scaffold is still missing. The objective of this study was to investigate the in vitro and in vivo regenerative capacity of graphitic carbon nitride (g-C_3_N_4_) and graphene oxide (GO) nanomaterials to stimulate critical-sized bone defect regeneration. The in vitro cytotoxicity and hemocompatibility of g-C_3_N_4_ and GO were evaluated, and their potential to induce the in vitro osteogenesis of human fetal osteoblast (hFOB) cells was assessed using qPCR. Then, bone defect in femoral condyles was created in rabbits and left empty as control or filled with either g-C_3_N_4_ or GO. The osteogenesis of the different implanted scaffolds was evaluated after 4, 8, and 12 weeks of surgery using X-ray, computed tomography (CT), macro/microscopic examinations, and qPCR analysis of osteocalcin (OC) and osteopontin (OP) expressions. Both materials displayed good cell viability and hemocompatibility with enhanced collagen type-I (Col-I), OC, and OP expressions of the hFOB cells. Compared to the control group, the bone healing process in g-C_3_N_4_ and GO groups was promoted in vivo. Moreover, complete healing of the bone defect was observed radiologically and grossly in g-C_3_N_4_ implanted group. Additionally, g-C_3_N_4_ implanted group showed higher percentages of osteoid tissue, mature collagen, biodegradation, and expressions of OC and OP. In conclusion, our results revealed that g-C_3_N_4_ and GO nanomaterials could induce osteogenesis in critical-sized bone defects.

## Introduction

Critical-sized bone defects have been reported in millions of patients each year due to massive bone loss associated with violent trauma, blast injuries, excision of bone tumors, and skeletal malformations^1,2^. Although the intrinsic bone healing capacity, bone defect regeneration is impaired when the gap of bone loss exceeds the critical size. Therefore, critical-sized bone defect repair is considered a significant obstacle in orthopedics and represents an important health issue with economic implications^3^.

Autogenous and allogenic bone implantation has been utilized widely to reconstruct the critical-sized bone defects; however, their clinical application has several restrictions. Autogenous bone implantation is usually associated with limited supply, post-operative pain, blood loss, morbidity of the donor site, and prolonged period of operation. Allogenic bone grafts are vulnerable to the risk of disease transmission, poor osseointegration, and rejection^3–5^. These hurdles inspired the development of innovative alternative therapies based on tissue engineering to stimulate and support bone formation^2,6^. Bone tissue engineering includes a combination of implanted cells, cytokines, and/or biodegradable scaffolds. Scaffolds have an integral role in bone regeneration as they provide an extracellular microenvironment that supports cell proliferation and differentiation. Besides, the properties of the ideal scaffolds include excellent biocompatibility, biodegradability, porosity, and mechanical strength^6–10^.

Different biomaterials have been studied over the last years as a scaffold for bone tissue engineering to repair the critical-sized bone defects; however, none of them is considered ideal^3–7,11^. Among different biomaterials, carbonaceous nanomaterials (CNs) have been used increasingly in various biomedical applications in the last years^12^.

Graphitic carbon nitride (g-C_3_N_4_) is a CNs with unique optical and electronic properties, low-cost, straightforward synthesis procedures, physicochemical stability, excellent biocompatibility and biometabolizability, and novel fluorescent characteristics^13^. The g-C_3_N_4_-based nanocomposites have been reported as promising materials for biomedical applications, including tissue regeneration^14,15^. It has been reported that photoactivated C_3_N_4_ induces and supports the in vitro osteogenic proliferation and differentiation. In addition, it activates Runt-related transcription factor 2 (Runx2) that promotes the expression of osteoblast marker genes^15^.

Graphene oxide (GO) is another CNs that has recently emerged in the biomedical field for scaffold fabrication in tissue engineering^7,11,16–18^, drug delivery^19,20^, gene therapy^21,22^, cancer therapy^23,24^, wound healing^25^, and antibacterial^26,27^ as well as antiviral applications^28,29^ due to their unique physicochemical and mechanical properties such as high thermal conductivity^30^, high drug loading efficiency^31^, and water dispensability^32^ as well as biocompatibility^33^ and biodegradability^34^. GO and ultrasonicated GO have been reported to provide a favorable platform that enhance and support mesenchymal stem cells (MSCs) adhesion, proliferation, and differentiation into osteogenic lineage cells as well as osteoblast mineralization^7,11,16,35,36^. Besides, it has been suggested that the hydrophobic π domains in GO structure improve its interactions with proteins through hydrophobic and electrostatic interactions, therefore GO can induce stem cells differentiation into osteogenic cells^37–39^.

Thus, the main goal of this study is to evaluate the osteo-regenerative efficiency of g-C_3_N_4_ and GO using in vitro and in vivo studies in a rabbit femoral condyle model. Additionally, it aims to investigate the in vitro and in vivo biocompatibility of g-C_3_N_4_ and GO scaffolds.

## Results

### Characterization of g-C_3_N_4_ and GO nanomaterials.

**Figure 1:**
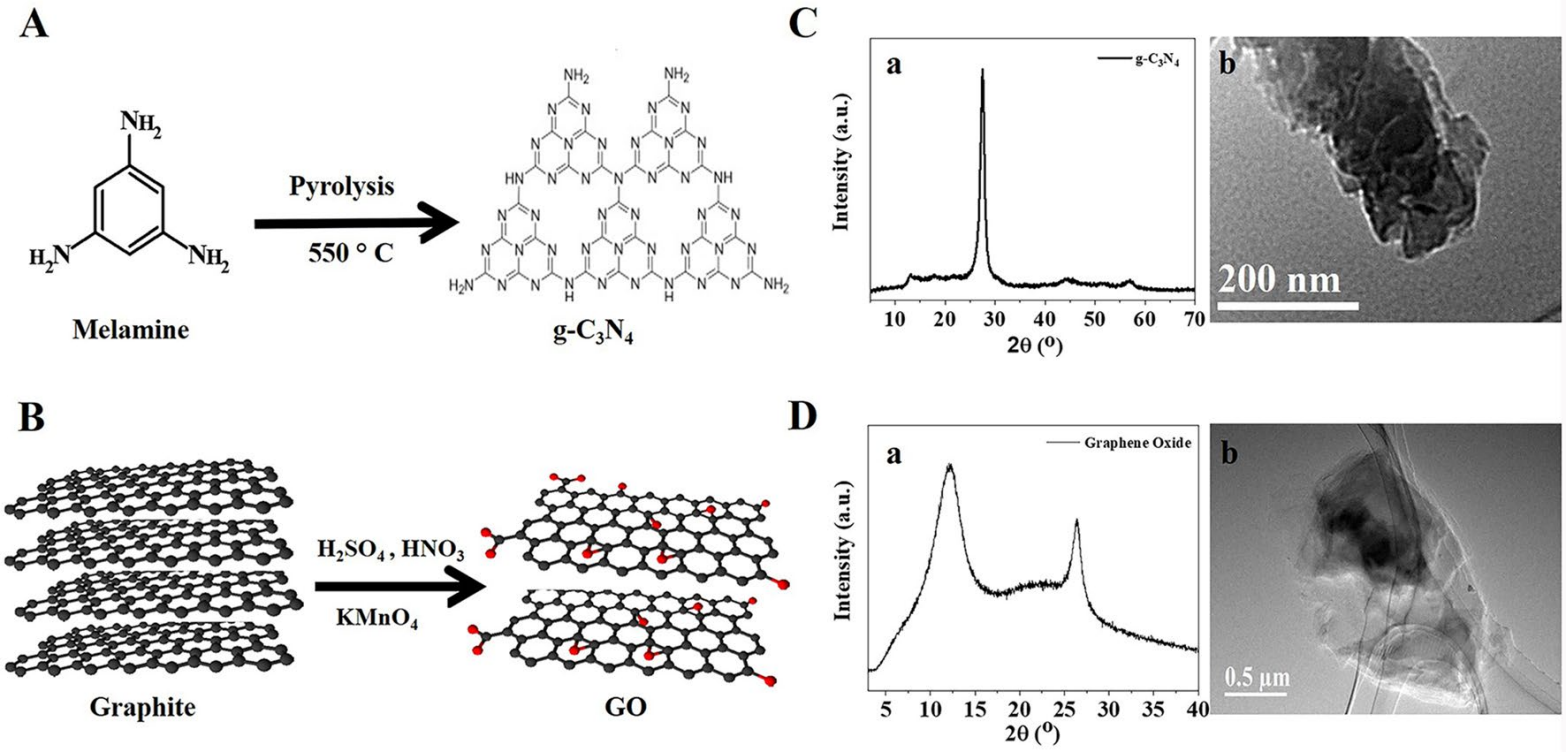
Synthesis and characterization of g-C_3_N_4_ and GO. (**A**) Schematic representation for the synthesis of g-C_3_N_4_ and their characterization (**C**) using XRD (a), and TEM (b). (**B**) Schematic representation for the synthesis of GO and their characterization (**D**) using XRD (a), and TEM (b).

The synthesis and characterization for g-C_3_N_4_ and GO nanomaterials are plotted in Fig. 1. As shown in Fig. 1A, the polymerization of melamine at 550^o^ C resulted in a yellow g-C_3_N_4_ powder. The XRD pattern of g-C_3_N_4_ displayed a sharp peak located at 2θ ≈ 27.4° with a distance (*d*) spacing of ∼0.33 nm, corresponding to the periodic stacking of the conjugated aromatic layers (Fig. 1Ca). TEM imaging of g-C_3_N_4_ showed a layer stacking of g-C_3_N_4_ layers^40^ (Fig. 1Cb).

Hummers’ method produced a black GO powder (Fig. 1B). XRD pattern of GO showed a diffraction peak at 2θ of 12.0° (d-space = 0.78 nm) and 26.5° (d-space = 0.33 nm) for Miller planes of (001) and (002) for GO and graphite, respectively^41,42^ (Fig. 1Da). TEM imaging of GO showed a transparent particle indicating the formation of a few GO layers (Fig. 1Db). Zeta potential of GO colloidal solution at pH 7 was − 45 mV. The high negative zeta potential is due to the oxygen functional groups such as hydroxyl, carbonyl, carboxylate, and epoxide^43^. The presence of oxygen functional groups enables high dispersion of GO in water. Raman spectrum shows the main characteristic peaks at 1348 cm^–1^ and 1598 cm^–1^, corresponding to D (vibration of the *sp*^3^ carbon atoms), and G (vibration of the *sp*^2^ carbon atoms), respectively^44^ (Fig. supp 1A). The intensity of I_D_/I_G_ ratio equals 0.96, which is close to the previous value reported for GO (0.97)^45^, indicating multilayer structure. SEM image and elemental analysis using EDS were reported as shown in Fig. supp 1B. The layer morphology of GO can be noticed from SEM image. EDX analysis shows the presence of 25.4 wt.% oxygen, revealing that the carbon-to-oxygen atomic ratio is 2.8, close to the value reported using Hummer’s method^43,46^.

### Indirect contact cytotoxicity assay

**Figure 2:**
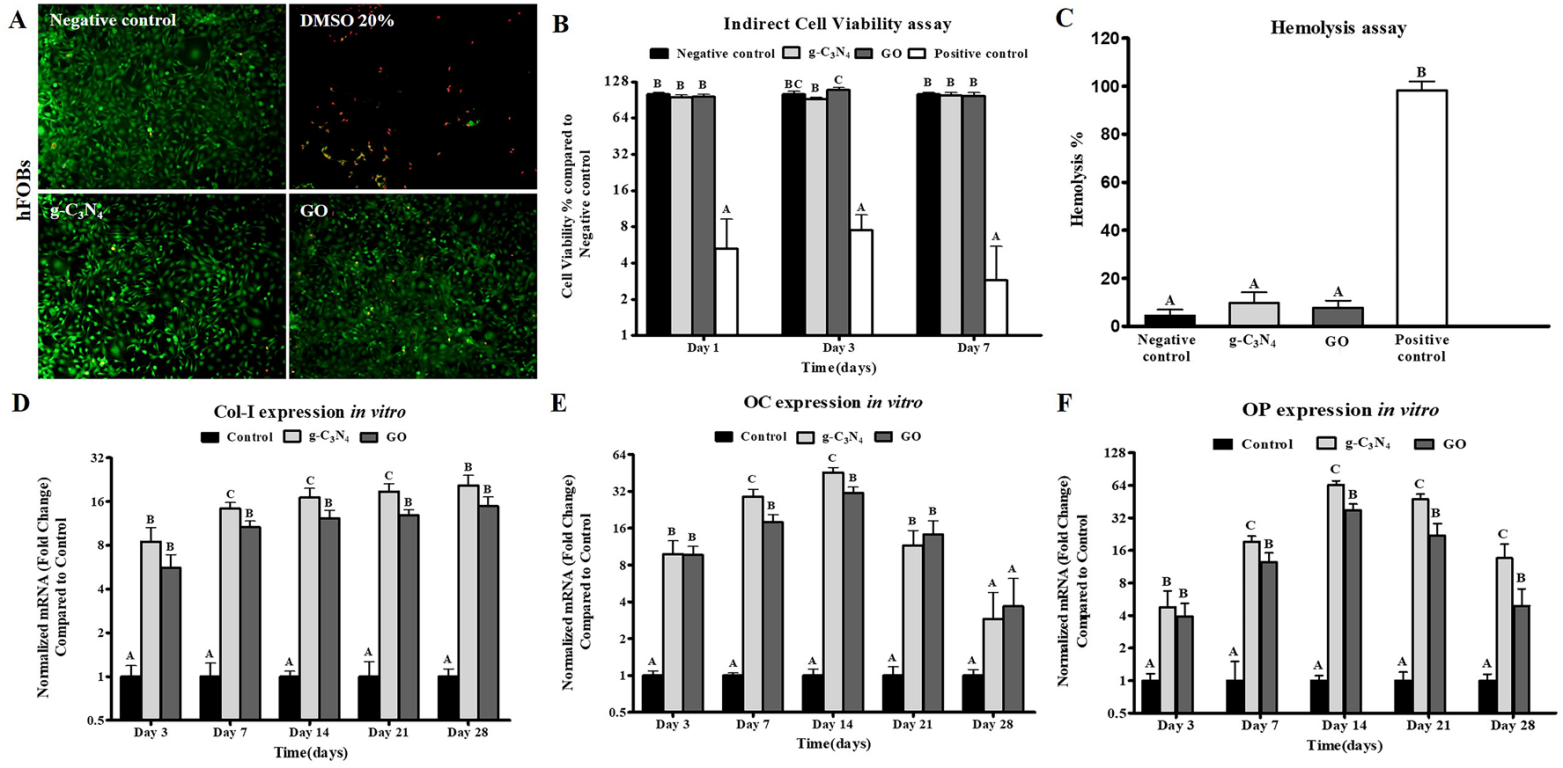
In vitro experiments of g-C_3_N_4_ and GO. (**A**) Cell viability using Live/Dead assay for hFOB cells cultured on g-C_3_N_4_ and GO nanomaterials for 7 days. Live cells were stained green and dead cells were stained red (Scale bar represents 100 µm, Magnification = 10 ×). (**B**) MTT cytotoxicity assay for cell viability of hFOB cultured using extracts of g-C_3_N_4_ and GO nanomaterials for 1, 3 and 7 days compared to the negative control. Error bars represent means ± standard deviation (n = 8). (**C**) Hemolysis assay using extracts of g-C_3_N_4_ and GO nanomaterials compared to the negative control. q-PCR analysis for mRNA expression of collagen type 1 (Col-1) (**D**), osteocalcin (OC) (**E**), and osteopontin (OP) (**F**) in negative control cells and on g-C3N4 and GO nanomaterials for 28 days, respectively. Error bars represent means ± SD; n = 3 for each group and time point. Bars with the same letter represent not significantly different values (one-way ANOVA followed by Tukey's HSD post hoc test).

The live/dead staining assay revealed that all the preconditioned media from the different nanomaterials revealed only a few positive cells to Ethidium Homodimer-1 staining after 7 days of culture (Fig. 2A).

The proliferation of hFOB cells was evaluated using extraction media prepared from the different samples. On day 1, preconditioned media prepared from both nanomaterials showed viability of more than 95% compared to the negative control by MTT assay (*P* < 0.05) (Fig. 2B). By day 3, preconditioned media prepared from GO nanomaterial revealed higher viability than g-C_3_N_4_ nanomaterial with percentages of 108.74% ± 6.32% compared to 91.7% ± 2.87% in g-C_3_N_4_ (*P* < 0.05). Finally, no significant difference was observed between the different materials on day 7.

### Hemocompatibility evaluation

The g-C_3_N_4_ and GO nanomaterials displayed a non-significant hemolysis rate of 9.64 ± 4.58% and 7.61 ± 3.14%, respectively, whereas the negative control group (PBS group) had a hemolysis rate of 4.35 ± 2.58% (*P* < 0.05) (Fig. 2C).

### qPCR analysis

The expression of Col-1 in cells cultured on g-C_3_N_4_ and GO displayed a significantly higher level than the negative control group starting from day 3 till day 28 (Fig. 2D). On day 3 and day 28, no significant difference between the designed nanomaterials was observed. However, Col-1 expression was significantly higher in g-C_3_N_4_ nanomaterial than in the GO nanomaterial on day 7, day 14, and day 21 with fold changes of 14.34 ± 2.17-fold, 17.09 ± 1.5-fold, and 18.73 ± 2.32-fold, compared to 10.66 ± 1.11-fold, 12.31 ± 1.62-fold, and 12.83 ± 1.22-fold in cells grown on GO, respectively (*P* < 0.05).

OC expression was higher in cells cultured on the designed nanomaterials than in the negative control group (Fig. 2E). On days 7 and 14, hFOB cultured on g-C_3_N_4_ nanomaterials showed a significantly higher expression than GO nanomaterials (*P* < 0.05). Starting from day 21, the expression of OC decreased gradually till reaching a non-significant level of expression with that in the negative control group at day 28.

Finally, OP expression was significantly higher in the g-C_3_N_4_ and GO groups at the different evaluation times compared to the negative control group (Fig. 2F). On days 7, 14, 21, and 28, cells on g-C_3_N_4_ nanomaterial expressed a higher expression of OP (19.25 ± 2.42, 64.67 ± 5.98, 47.72 ± 5.40, and 13.57 ± 4.70-folds, respectively) compared to GO nanomaterial (12.46 ± 2.71, 37.64 ± 5.39, 21.95 ± 6.48, and 4.92 ± 2.13-folds, respectively) (*P* < 0.05).

### Clinical observation

The critical size bone defect was successfully created, as demonstrated in Fig. supp 2. All rabbits survived during and after surgery. They recovered from anesthesia within 30–45 min after surgery and could stand up and move freely within the first 24 h after the operation. They returned to normal activities such as eating, drinking, and grooming within 48 h after surgery. There were neither operative nor postoperative complications such as infection and fracture were recorded. The wound healing was uneventful without dehiscence during the postoperative period for all groups, and the sutures were removed 7–10 days after the surgical operation.

### Radiographical assessment

**Figure 3:**
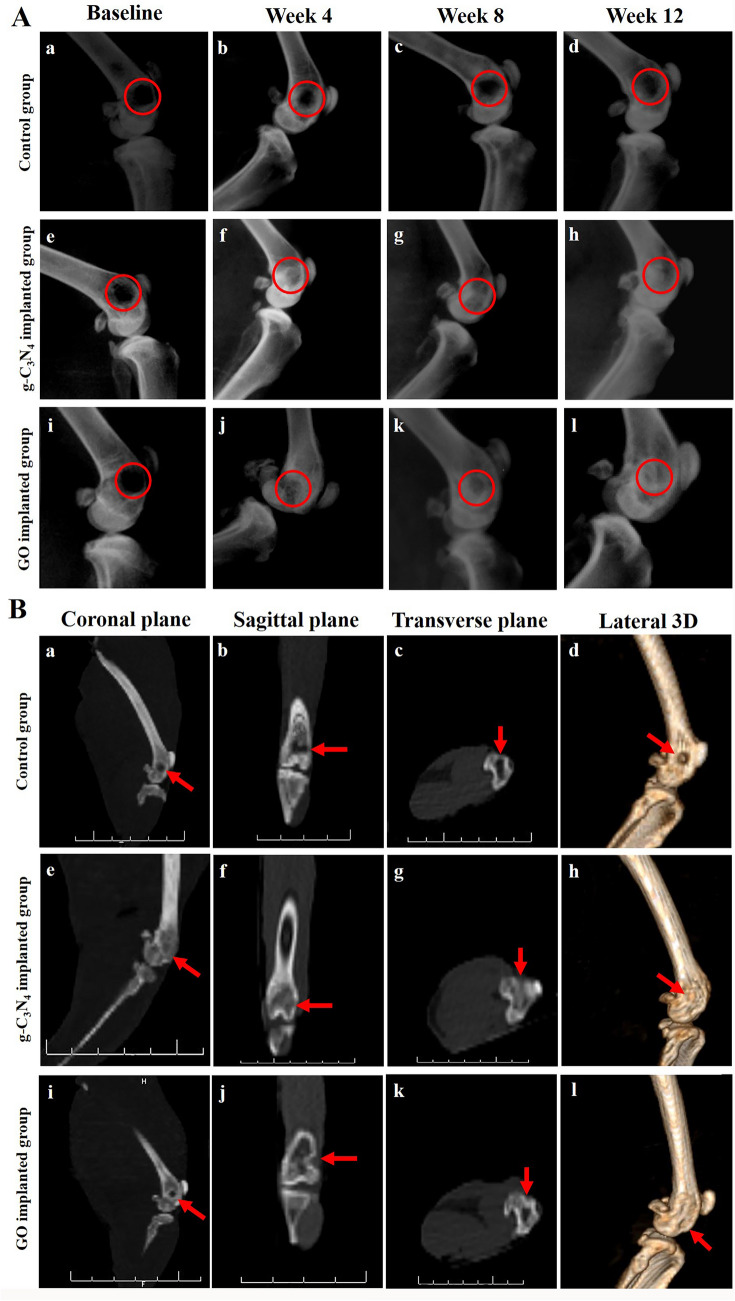
Radiological evaluation of bone repair. (**A**) Lateromedial radiographs of the defect site of control (a–d), g-C_3_N_4_ implanted (e–h), and GO implanted (i–l) groups at different post-implantation times. (**B**) CT scanning of defect site of control (a–d), g-C_3_N_4_ implanted (e–h), and GO implanted (i–l) groups at week 12 after surgery.

The immediate postoperative radiographs revealed well-defined radiolucent defects in the femoral condyles in different groups of this study (Fig. 3Aa,e,i).

On week 4 after surgery, a well-defined radiolucent defect was already visible in the control group (Fig. 3Ab). However, g-C_3_N_4_ and GO implanted groups showed increased radiopacity of the bone defects over time (Fig. 3Af,j). The defect sites were more radiopaque than immediate postoperative radiographs in both g-C_3_N_4_ and GO implanted groups. g-C_3_N_4_ implanted defects showed new bone formation at the center of these defects with hardly distinct margins, while in GO implanted defects, the new bone formation could be detected within the defects; however, their margins were still clearly distinguishable.

On week 8 after surgery, the control group still displayed a well-demarcated radiolucent defect (Fig. 3Ac). However, in the g-C_3_N_4_ implanted group, most defects were filled with newly formed bony tissue and markedly indistinct from the surrounding bony tissue (Fig. 3Ag). In addition, in GO implanted group, there was evidence for more bone formation at these defects with hardly distinct margins (Fig. 3Ak).

On week 12 after surgery, the control defects still appeared radiolucent with a small amount of bony tissue formation at their margins (Fig. 3Ad). However, g-C_3_N_4_ and GO implanted defects were nearly indistinguishable from the adjacent bone and almost as dense as normal bone (Fig. 3Ah,l).

The bone density according to the mean grayscale value per unit area of defects on week 4 after surgery was significantly higher in the g-C_3_N_4_ implanted group (2861.34 ± 157.9) than in both GO implanted (2218.93 ± 88.2) and control groups (1932.5 ± 226.9) (*P* < 0.05) (Fig. supp 3). However, on week 8 and week 12 after surgery, bone density was significantly higher in both g-C_3_N_4_ (3203.5 ± 225.02 and 3305.4 ± 160.9, respectively) and GO (2924.7 ± 221.8 and 3259.4 ± 561.9, respectively) implanted groups than the control group (2049.6 ± 63.9 and 2172.5 ± 40.7, respectively) (*P* < 0.05). Furthermore, a non-significant difference was observed between the various implantation time points within the control or g-C_3_N_4_ implanted groups. In contrast, the GO implanted group displayed a significant difference between week 4 and both weeks 8 and 12 after implantation (*P* < 0.05).

### Computed tomography (CT) scanning

No bone union could be detected in the coronal, sagittal, and transverse planes of the control group (Fig. 3Ba–c). However, bone union was detected in g-C_3_N_4_ and GO implanted defects. The g-C_3_N_4_ implanted group revealed that the bone defects filled with newly formed bone tissue in the different planes (Fig. 3Be–g), while the GO implanted group showed the presence of islands of newly formed bone within the bone defects (Fig. 3Bi–k).

The results of the different planes were correlated to lateral 3D-CT images (Fig. 3Bd,h,l) whereas bone defects appeared undistinguished with smooth cancellous bone union in g-C_3_N_4_ implanted group, and were less detectable in GO implanted group.

### Gross examination of the bone defects

As shown in Fig. supp 4, the margins of the defects at different evaluation periods were demarcated in the control group. On week 4 after surgery, the g-C_3_N_4_ implanted group revealed less distinguishable defects margins, while GO implanted group showed a well-defined defects margin. However, the defects’ margins were indistinguishable in the g-C_3_N_4_ implanted group and less demarcated in the GO implanted group at week 8. The margins of the defects in the g-C_3_N_4_ implanted group at week 12 were indistinguishable, whereas GO implanted group still showed a less detectable defects margin.

The defects in the control group were filled with blood clots and/or connective tissue on week 4 and connective or fatty tissues on weeks 8 and 12 postoperatively.

The g-C_3_N_4_ implanted defects at week 4 were covered with the yellow particles of the g-C_3_N_4_ scaffold. In addition, the g-C_3_N_4_ material was well integrated within the bone defects and appeared binding to the host bone with indistinguishable interface between the scaffolds and host bone. However, bridging bone-like tissue connecting the rims of the bone defects with a little g-C_3_N_4_ material was observed on week 8. On week 12, bone defects were undistinguished with a smooth surface and a color resembling the surrounding tissue, indicating complete repair of the bone defects in the g-C_3_N_4_ implanted group with bridging bone-like tissue.

In GO implanted group, the defects at week 4 were covered with the black GO scaffold that was well integrated within the bone defects and appeared bound to the host bone with indistinguishable interface between the GO scaffold and host bone. However, on weeks 8 and 12, bone defects were still covered with the black-colored GO, and partial repair of the bone defects with a bridging smooth bone-like tissue connecting the rims of the bone defects was detected.

### Histological examination

**Figure 4:**
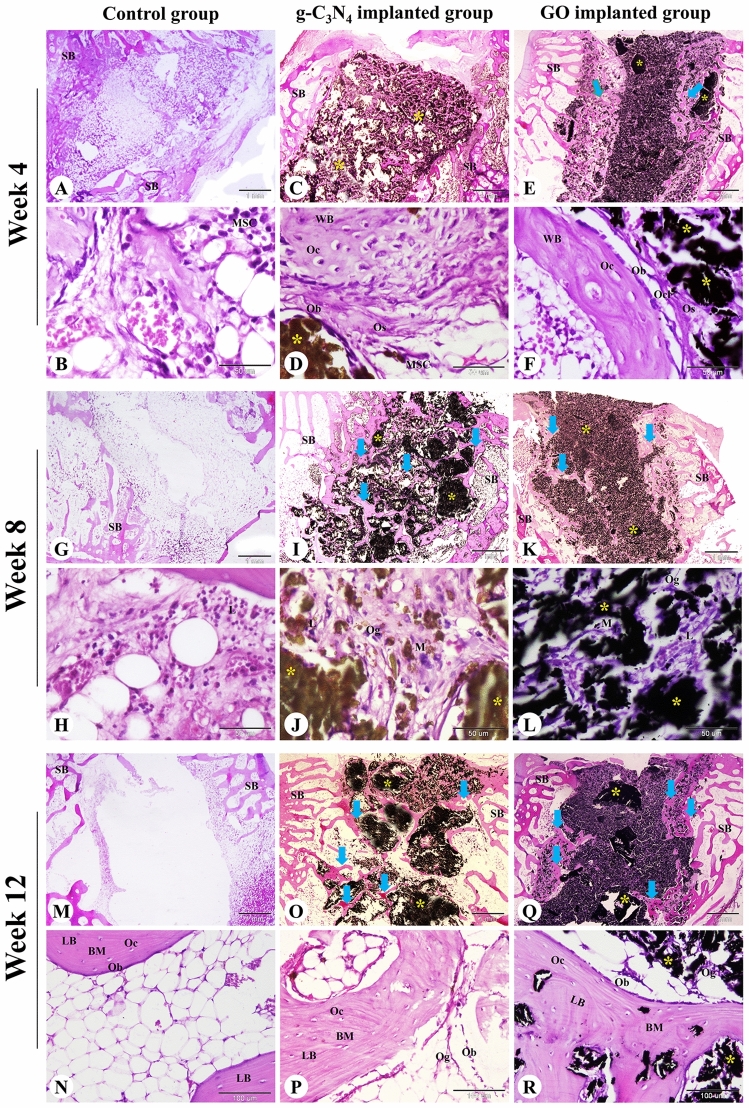
Histological evaluation of femoral condyle defects in rabbits. The repair site of the femoral condyle at week 4 (**A**–**F**), 8 (**G**–**L**), and 12 (**M**–**R**) after surgery in control, g-C_3_N_4_ implanted, and GO implanted groups was stained with H&E. *SB* spongy bone, *MSC* mesenchymal stem cell, *Ob* osteoblast, *Oc* osteocyte, *Ocl* osteoclast, *Og* osteogenic cells, *Os* osteoid tissue, *BM* bone matrix, *WB* woven bone, *LB* lamellar bone, *L* lymphocyte, *M* macrophage; yellow asterisks: implanted nanomaterial; blue arrows: newly formed spongy bone. The scale bars in panels (**A**, **C**, **E**, **G**, **I**, **K**, **M**, **O**, and **Q**) = 1 mm, panels (**N**, **P**, and **R**) = 100 μm, panels (**B**, **D**, **F**, **H**, **J**, and **L**) = 50 μm.

Histological evaluation of femoral condyle bone defects harvested on weeks 4, 8, and 12 after surgery was conducted to examine the effect of g-C_3_N_4_ and GO nanomaterials on bone repair. At week 4, the control bone defects were filled with fatty bone marrow containing abundant fat cells and a few hematopoietic stem cells and MSCs (Fig. 4A,B). However, the bone defect in the g-C_3_N_4_-implanted group was filled with disintegrated scaffold material separated by osteoid tissue, osteogenic cells, osteoblasts, osteoclasts, and collagen fibers and surrounded by spongy bone trabeculae (Fig. 4C,D). The direction of osteoregeneration was centrifugal. The peripheral zone of the bone defect displayed newly formed woven bone and some MSCs (Fig. 4C). The changes of the GO-implanted group were similar to the g-C_3_N_4_-implanted group, but the direction of osteoregeneration was centripetal and there was less newly formed woven bone with more scaffold material (Fig. 4E,F).

At 8 weeks, the control defects were still filled with fatty bone marrow (Fig. 4G,H). The bone defect in the g-C_3_N_4_ implanted group contained more regenerated bone tissues with less residual scaffold materials compared to the GO implanted group. The residual materials were surrounded and separated by osteoid tissue and an anastomosing network of newly formed woven bone trabeculae which annealed to the peripheral spongy bone. Moreover, lymphoid aggregation and neovascularization were observed in the implanted bone defect area (Fig. 4I–L).

At 12 weeks, the control group remained poorly repaired and bone formation could not be seen (Fig. 4M,N). However, more anastomosing networks of the newly formed woven and lamellar bone trabeculae could be seen in the implanted area and connected to the peripheral spongy bone in the g-C_3_N_4_ implanted group than that in the GO implanted group. Inflammatory cell infiltration and neovascularization were still seen in the implanted bone defect area. At the same time, the materials were further degraded (Fig. 4O–R).

**Figure 5:**
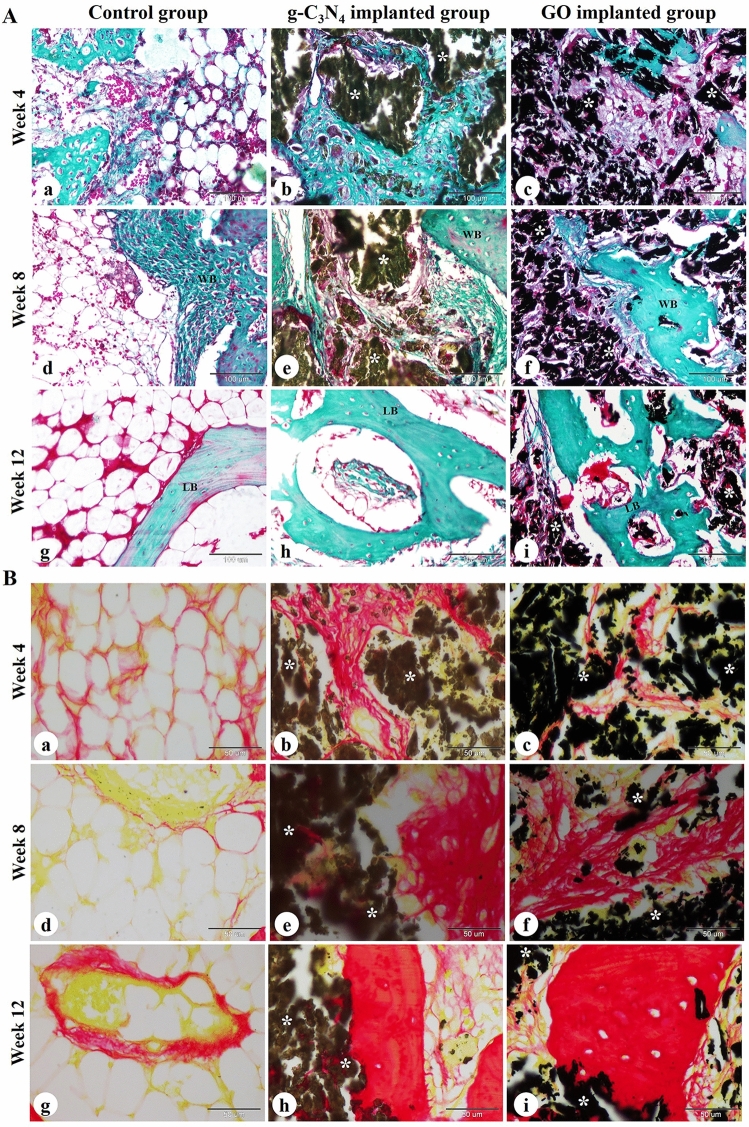
Histochemical evaluation of femoral condyle defect in rabbits. The repair site of the femoral condyle at weeks 4, 8, and 12 after surgery in control (a, d, g), g-C_3_N_4_ implanted (b, e, h), and GO implanted groups (c, f, i) was stained with Crossmon's trichrome (**A**) and Sirius red (**B**) stains. *WB* woven bone, *LB* lamellar bone; white asterisks: implanted nanomaterial. The scale bars in Crossmon’s trichrome stain panels = 100 μm, Sirius red stain panels = 50 μm.

The bone defects were further evaluated using Crossmon's trichrome and Sirius red staining (Fig. 5). The bone defects remained unrepaired in the center of the control group with the formation of newly formed bone containing mature collagen at the periphery. However, the deposition of mature collagen at the newly formed bone matrix was found in the central and peripheral regions of the bone defects in both g-C_3_N_4_ and GO implanted groups. Clearly, the amount of mature collagen in the newly formed bone was higher in g-C_3_N_4_ implanted group than the GO group (Fig. 5).

In addition, the defects were stained with PAS and hematoxylin (Fig. supp 5). The stained sections showed no chondrocytes. However, PAS-negative osteogenic cells, osteoblasts, and osteocytes in addition to PAS-positive bone matrix could be seen in the peripheral zone of all bone defects and the central zone of the implanted groups.

### Histomorphometric analysis:

**Figure 6:**
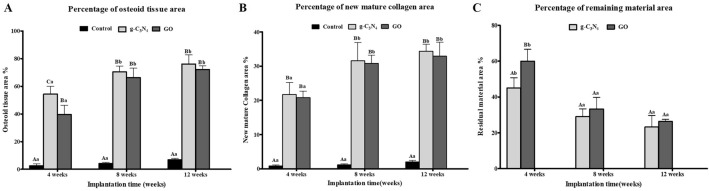
In vivo histomorphometrical analysis. The percentages of osteoid tissue area (**A**), residual material area (**B**), and new mature collagen area (**C**) to the total defect area in rabbit femoral condyle defects implanted with g-C_3_N_4_ and GO or left empty at various times post-implantation. Error bars ± SD; n = 3 for each group and time point. Bars with the same letter represent values that are not significantly different (two-way ANOVA followed by Tukey's HSD post hoc test). (**A**–**C**) significance between groups; a, b, and c: significance between time points within the same group.

In general, the Os% in the g-C_3_N_4_ and GO implanted defects displayed a highly significant difference (*P* < 0.05) compared with the control defect throughout the experiment (Fig. 6A). At 4 weeks, the Os% in g-C_3_N_4_ implanted defects (54.30 ± 5.71%) was significantly higher than in the GO implanted group (39.69 ± 6.56%) (*P* < 0.05). Both g-C_3_N_4_ and GO implanted groups displayed no significant difference at week 8 and week 12 after implantation (Fig. 6A).

The Col% in the defects implanted with g-C_3_N_4_ and GO were significantly higher than the control defects throughout the different implantation times (*P* < 0.05) (Fig. 6B). On weeks 4, 8, and 12 after implantation, the g-C_3_N_4_ implanted defects showed Col% of 21.69 ± 3.51%, 31.60 ± 5.29%, and 34.36 ± 2.07%, respectively compared to 20.84 ± 1.85%, 30.87 ± 2.33%, and 32.93 ± 4.04%, respectively in the GO group (Fig. 6B).

The RM% was significantly lower in g-C_3_N_4_ treated defects (45.07 ± 5.69%) than GO treated defects (59.98 ± 6.60%) at week 4 after implantation (*P* < 0.05). However, no significant differences were detected at weeks 8 and 12 after implantation of the nanomaterials (Fig. 6C).

Furthermore, in g-C_3_N_4_ and GO implanted groups, the Os% and Col% were significantly lower (*P* < 0.05) at week 4 after implantation compared to weeks 8 and 12. However, the RM% was considerably higher at week 4 after implantation than weeks 8 and 12 (*P* < 0.05) (Fig. 6).

### IHC of CD34

CD34 + mesenchymal stem cells were numerous and formed a network of interconnected cells surrounding the g-C_3_N_4_ implanted material, whereas they were less numerous in the GO implanted group (Fig. supp 6).

### qPCR analysis

**Figure 7:**
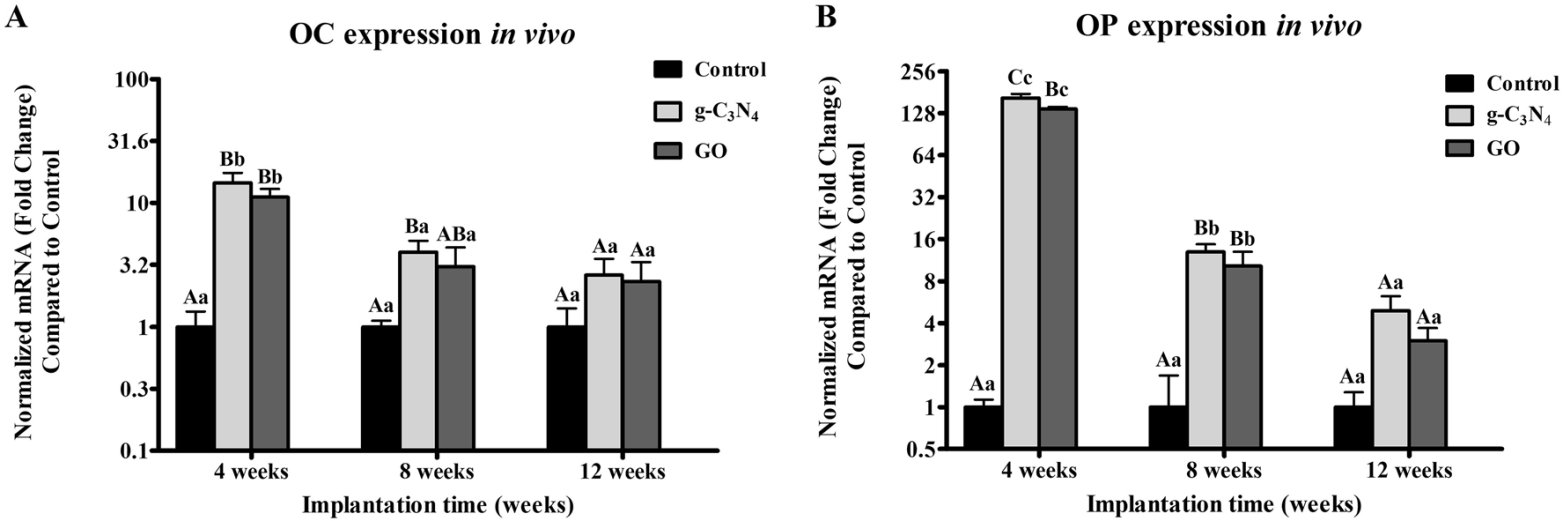
In vivo qRT-PCR analysis. q-PCR analysis for mRNA expression of osteocalcin (OC) (**A**) and osteopontin (OP) (**B**) in different groups at week 4,8, and 12 post-implantations. Error bars ± SD; n = 3 for each group and time point. Bars with the same letter represent values that are not significantly different (two-way ANOVA followed by Tukey's HSD post hoc test). (**A**–**C**) significance between groups; a, b, and c: significance between time points.

The mRNA expression of OC and OP in all implanted groups revealed a higher level on week 4 after implantation, then decreased dramatically over time. OC expression was higher in g-C_3_N_4_ and GO implanted defects than in the control group on week 4 after implantation (*P* < 0.05), while it displayed a significance between g-C_3_N_4_ implanted defects and control one on week 8 (*P* < 0.05) (Fig. 7A). However, g-C_3_N_4_ and GO implanted groups showed non-significant differences between each other at different evaluation times (Fig. 7A).

OP expression level was higher in g-C_3_N_4_ and GO implanted defects compared to that in the control group at week 4 and week 8 after implantation (*P* < 0.05) (Fig. 7B). Additionally, the g-C_3_N_4_ implanted group showed a significance with GO implanted group at week 4 post-implantation (*P* < 0.05) (Fig. 7B).

The expression levels of OC and OP of g-C_3_N_4_ and GO implanted defects were significantly higher at week 4 than weeks 8 and 12 after implantation. Moreover, OP expression was higher at week 8 after implantation than week 12 (Fig. 7).

## Discussion

As a health burden, critical-sized bone defects represent one of the leading causes of disability, resulting in a decline in life quality^2^. Bone tissue engineering is an emerging field that serves to construct bone substitutes to overcome the shortcomings of conventional treatments of bone defects^6^. Even though several studies have reported the fabrication of different tissue-engineered bone grafting scaffolds to accelerate the bone healing process, the ideal material has not been developed yet^3–5^. In the present study, we tested the biocompatibility and the bone regeneration capacity of g-C_3_N_4_ and GO scaffolds in vitro and in vivo.

Herein, g-C_3_N_4_ was synthesized via direct pyrolysis of melamine because it is a rapid and economical process^47^. While GO was synthesized in this study via exfoliation using the Hummer method, which is simple, low-cost, efficient, stable over 3–5 months, and environmentally friendly procedure^16^. The results of XRD revealed a sharp reflection peak at the position of 27.4° (*d*∼0.33 nm) that matches the predicted (2θ) diffraction of g-C_3_N_4_. This finding is similar to the XRD patterns of g-C_3_N_4_ reported in other studies^15,47,48^. In the diffractogram of GO, the diffraction peak at Bragg angle 12.0° refers to the Miller index (002), whereas the diffraction peak at 26.5° refers to residual graphite or re-stacking layers of the exfoliated GO^16,17^.

The cytocompatibility of the designed biomaterials is a key criterion to confirm their ability to support the host bone cell growth and adhesion for in vivo orthopedic applications^7^. Hence, in this study hFOB cells have been cultured on extracts of g-C_3_N_4_ and GO nanomaterials to test their cytotoxicity^16^. Our results revealed a non-toxic effect of g-C_3_N_4_ and GO nanomaterials on hFOB cells, indicating that they are cytocompatible and their use as orthopedic scaffolds would not affect the normal physiological microenvironment after in vivo implantation. These findings are in agreement with Tiwari et al.^15^ and Hussein et al.^16^. Tiwari et al.^15^ reported high viability of HeLa cells upon culture on C_3_N_4_, for 24 h, while Hussein et al.^16^ showed good viability of human endothelial cells, hFOB cells, and mouse embryonic fibroblasts after seeding on GO for 7 days. Since the biomaterial would come in contact with blood directly after implantation, hemocompatibility testing is critical. In the current study, both g-C_3_N_4_ and GO nanomaterials displayed a non-hemolytic effect, indicating excellent hemocompatibility of the designed nanomaterials as reported previously^16,49^.

The potential of biomaterials to enhance bone cell function is another key criterion in bone tissue engineering. Both g-C_3_N_4_ and GO showed a significant upregulation of Col-I, OC, and OP expressions, whereas the g-C_3_N_4_ nanomaterial showed the highest expression of the osteogenic proteins especially in the early stages of healing. These proteins play an essential role in osteoblast proliferation and differentiation, bone mineralization, and bone remodeling^50–55^. Collectively, our data indicated that the designed nanomaterials could stimulate the osteoblast’s biological activities and functions in vitro*.* This observation may be attributed to the material’s ability to induce the expression of Runx2 that interacts with the promoter regions of osteoblast-specific genes, including Col-I, OC, and OP^15,37,56^. Interestingly, a sharp decline in OC and OP expression on day 21 was observed, which may be caused by the complete mineralization of cells, as suggested previously^57–60^.

To further confirm the in vitro results, we implanted the designed materials in a well-established critical-sized femoral condyle bone defect model (Ø 5 × 10 mm) in rabbits^61,62^. In this study, only male rabbits were used for investigating the in vivo behaviour of the materials to avoid the influence of female sex hormones on bone healing^63,64^. Additionally, males are superior to females in bone regeneration due to the higher MSCs quantity as reported previously^63^. Moreover, Pien et al., recorded a better osseointegration with larger peri-implant bone volume after 30 days in male rats compared to females^65^. The interaction between the host cells and the implanted materials activates the osteogenesis process. The implantation of biomaterials causes a foreign body response, which is characterized by infiltration of different inflammatory cells and fibrosis of the surrounding tissues^8,66^. However, our results showed mild infiltration of the inflammatory cells, e.g., macrophages, in both g-C_3_N_4_ and GO implanted groups, confirming their high biocompatibility and favorably creating an osteogenic milieu that can improve osteogenesis. Inflammatory cell infiltration, particularly macrophages, is crucial for the biodegradation of the implanted materials^67^. Our results showed that both g-C_3_N_4_ and GO were biodegraded gradually over time. However, the g-C_3_N_4_ implanted defects displayed the lowest percentage of residual materials.

In addition, growth factors such as bone morphogenetic proteins (BMPs), transforming growth factor β (TGF-β), and vascular endothelial growth factor (VEGF), and cytokines such as tumor necrosis factor-α (TNF-α), interleukin 1-β (IL1-β), IL-6, and IL-10, are secreted by macrophages^8,11,66^ to stimulate the osteogenesis process via migration of the undifferentiated MSCs with osteogenic potential from the bone marrow and peripheral blood and eventually differentiation, osteoblast maturation, collagen organization and mineralization^8,66,68,69^. MSCs migrated through the disintegrated scaffold materials to the center of the defect have a crucial role in bone regeneration and remodeling by providing a favorable environment with the ability to differentiate into osteoblasts and stimulate the production of several growth factors that promote the osteogenesis process^70–74^. Therefore, osteogenesis has been promoted in g-C_3_N_4_ and GO implanted groups, where MSCs differentiated to osteogenic cells and then to osteoblasts. Osteoblasts synthesize and secrete bone collagen and bone matrix, then transform into osteocytes. Finally, the newly formed woven bone remodels into lamellar bone^75,76^.

Our results showed the formation of immature woven bone, which was remodeled later to mature lamellar bone in the g-C_3_N_4_ and GO implanted defects. However, the new bone formation was significantly higher in the g-C_3_N_4_ group than in the GO. These differences are attributed to the properties of the materials including chemical composition, structure, and porosity. g-C_3_N_4_ is a polymeric material containing elements of C, N, and H, while GO is 2D materials of C, O, and H. Compared with the GO, g-C_3_N_4_ has electron-rich materials with basic surface properties. It offers H-bonding motifs enabling hydrogen bond formation with biomolecules inside the bone. The functional properties of g-C_3_N_4_ exhibit higher interconnectivity to proteins helps in the transfer of nutrients and elimination of metabolic waste, making the scaffold a more conducive milieu for MSCs attachment, growth, and proliferation^8^. Moreover, CD^34+^ cells were detected surrounding the implanted material. CD^34+^ was reported as a common marker for MSCs that can differentiate into osteoblasts in vitro^72,77,78^.

For investigating the in vivo capability of the designed nanomaterials to promote de novo bone formation at a molecular level, the expressions of osteogenic genes (OC and OP) were analyzed. Our results demonstrated upregulated expressions of these genes in both implanted groups. The power of the fabricated nanomaterials for OC and OP upregulation suggested to be related to their potential to induce canonical Wnt signaling^15,37^. The functional groups in g-C_3_N_4_ and GO stimulate the ligand Wnt3a to bind with the receptor lipoprotein receptor-related proteins-5 (LRP5), followed by an increase the intracellular Axis inhibition protein-2 (AXIN-2) and Catenin Beta (CTNNB) gene expressions leading to upregulation of the osteogenic regulators (Runx2, Distal-less homeobox 5 (Dlx5), and Osterix (Osx)) and consequently enhanced the osteogenesis^15,37,79^. Notably, mRNA expressions of OC and OP in the g-C_3_N_4_ and GO implanted groups displayed abrupt downregulation at weeks 8 and 12 after implantation; this might be related to the normal decrease in the production of bone matrix proteins in the late stages of bone healing^80,81^. Moreover, the overexpression of OP on week 4 after implantation in the different implanted groups may be attributed to its secretion by macrophages and osteoblast lineage cells^82^.

In conclusion, the g-C_3_N_4_ and GO are biocompatible nanomaterials with the potential to upregulate osteoblast’s marker genes in vitro in hFOBs. They provide a suitable in vitro and in vivo environment for stimulating cellular migration, proliferation, adhesion, and differentiation. In addition, they have a robust anabolic effect on the regeneration process of critical-sized bone defect in rabbits. Lastly, our data suggest the possibility to use g-C_3_N_4_ and GO biomaterials as promising bone substitutes for reconstructing the osseous defects that cannot heal naturally.

In the current study, it is worth mentioning that there were few limitations such as the use of small sample size and insufficient characterization of the materials in terms of porosity and biodegradation. Future studies should focus on the biomechanical characteristics and biodegradation of g-C_3_N_4_ and GO nanomaterials as promising scaffolds for bone tissue regeneration. In addition, the fate of g-C_3_N_4_ and GO degradation byproducts must be investigated in further studies. Moreover, future research is needed to be conducted on a large scale to compare the bone regenerative capacity of both g-C_3_N_4_ and GO nanomaterials relative to other previously well-known established scaffolds such as tricalcium phosphate, demineralized bone matrix, and hydroxyapatite.

## Materials and methods

### Materials

Melamine was purchased from Acros organics (Belgium) with a purity of ≥ 98%, and flake graphite (average particle diameter of 20 mm, 99.95% purity) was purchased from Alfa Aeser (Germany). Nitric acid (69–72%) and sulfuric acid (96.0%) were purchased from ElNaser company (Egypt).

### Synthesis of g-C_3_N_4_

The g-C_3_N_4_ was synthesized via the pyrolysis of melamine^15^. Typically, 10 g of melamine was placed in a crucible with a cover. The crucible was then placed in a muffle furnace and heated to 550 °C for 5 h with a heating rate of 2 °C/min under atmospheric nitrogen pressure. After the heating reaction, the crucible was left to cool down to room temperature in the furnace. The product was washed with absolute ethanol and distilled deionized water (3 × 50 mL) and dried at 80 °C for 24 h. A solution (1 mg/mL) of the designed material was prepared via ultrasonication dispersion overnight.

### Synthesis of GO

GO was prepared using a modified Hummers method^16^. Briefly, a mixture of nitric acid (10 mL) and sulfuric acid (15 mL) was added to graphite (1 g). The solution flask was soaked in ice to keep the temperature below 0 °C. Three grams of potassium permanganate were added into the reaction mixture that was stirred for 12 h. Then, hydrogen peroxide (30–32%, 15 mL) was added dropwise to remove the excess permanganate. The material was filtered and washed several times with water and diluted HCl to remove any metals. The GO (20 mg) solution was prepared via ultrasonic dispersion in water (20 mL).

### Materials characterization

The morphology and size of these nanomaterials were studied using a transmission electron microscope (TEM; JEM-2100, JEOL, Japan). The phase purity of the prepared materials was characterized using X-ray powder diffraction (XRD; Philips 1700 diffractometer, Germany) with a Cu K_α_ radiation diffractometer. Scanning electron microscopy (SEM) and energy-dispersive X-ray spectroscopy (EDS) were recorded by TM3000 (Hitachi, Japan). Raman spectrum for GO dispersion was measured using the Horiba Labram HR system (wavelength of 785 nm and power of 150 mW). The zeta potential of the GO colloidal was evaluated using Zetasizer (Malvern, UK).

### Indirect contact cytotoxicity assay

Extracts of materials were prepared to evaluate the potential toxic risk of leaching chemicals. The extracts of the g-C_3_N_4_ and GO were prepared via incubating samples in the serum-free 1:1 mixture of Ham’s F12 and Medium Dulbecco Modified Eagle’s minimal essential medium (DMEM) supplemented with 1% penicillin/streptomycin (p/s, Gibco; Grand Island, NY, USA) culture medium under the condition of 37 °C/120 r/min for 72 h, according to a ratio standard of 0.2 g/mL of culture medium^67^. The supernatant was withdrawn and centrifuged to prepare the conditioned extracts, then filtered using 0.4 μm filters and stored at 4 °C till performing the cytotoxicity test. Human fetal-osteoblast cell line (hFOB 1.19; American Type Culture Collection (ATCC), USA) was cultured in a 1: 1 mixture of Ham’s F12 and DMEM supplemented with 10% fetal bovine serum (FBS, Hyclone; Logan, UT, USA) and 2.5 mM l-glutamine (Gibco) and 1% p/s in a humidified incubator at 34 °C and 5% CO_2_. At 70% confluency, the cells were harvested via trypsinization. The cells were placed at a density of 15 × 10^3^ in a 48-well plate for 24 h using the complete culture medium, then the medium was aspirated, followed by the addition of 500 µL conditioned or control medium after adding 10% FBS. In the negative control group, cells were cultured with complete medium only, while the cells were cultured in the presence of 20% dimethyl sulfoxide (DMSO) in the positive control. The cell response against the extracts was evaluated after incubating the plate for 1, 3, and 7 days. To measure the metabolic activity of cells, [3-(4,5-dimethylthiazol)-2-yl]-2,5-diphenyltetrazolium bromide (MTT) assay was performed. Briefly, 50 μL of MTT solution (5 mg /mL, Sigma-Aldrich, St Louis, MO, USA) was added to each well and incubated at 37 °C for 4 h. After discarding the medium containing MTT, 250 μL DMSO was added to all wells to dissolve the formazan into a purple solution. After 10 min of incubation, 100 μL aliquots from the wells were pipetted into another 96-well plate. The color developed was quantified by recording the absorbance at a wavelength of 570 nm with a spectrophotometer. The cell activity was represented as the percentage of activity expressed by cells compared to the negative control.

To qualitatively assess the cell viability after 7 days, staining with a Live/Dead assay kit (calcein-AM/ethidium Bromide homodimer, Invitrogen, USA) according to the manufacturer’s instructions, then imaged using a fluorescence microscope (Olympus, Tokyo, Japan).

### Hemocompatibility evaluation using hemolysis assay

The hemolysis that may occur due to contact of materials with blood was measured according to Momtahan et al.^83^. Briefly, 10 mL of fresh blood was collected from 7 dogs and directly transferred to the laboratory. Erythrocytes were separated by centrifuging at 2000×*g* for 15 min, followed by dilution in 1 × phosphate buffer saline (PBS) to create an erythrocyte suspension with 2 × 10^9^ cells/mL. A sample of each nanomaterial was placed in a glass tube containing 5 mL of erythrocyte suspension and kept at room temperature on a shaker with 125 rpm for 60 min. Afterward, 1 mL of the suspension was collected from each tube and centrifuged for 3 min at 3000×*g*. Erythrocyte suspension without any specimens was utilized as a negative control, whereas tubes containing 25 mg of sodium dodecyl sulfate (SDS) added to the erythrocytes were used as positive controls. Finally, the absorbance of the supernatant was examined at a wavelength of 545 nm using a spectrophotometer, and the hemolysis percentage was calculated as in the equation:

$$Hemolysis (\%) = \frac{S545-N545}{P545-N545}\times 100,$$

where the absorbance for samples, negative control, and positive control were represented by S545, N545, and P545, respectively.

### Quantitative polymerase chain reaction (qPCR) analysis

The nanomaterials were placed into 48-well plates to investigate the ability of the materials to enhance the attachment of hFOB 1.19 cells. Shortly, cell suspension containing 10 × 10^3^ cells (500 μL) was added on the surface of the powder, and the plate was incubated at 34 °C in 5% CO_2_ for 28 days. After 3, 7, 14, 21, 28 days of culture, qPCR analysis for collagen type-I (Col-I), osteocalcin (OC), and osteopontin (OP) expressions were performed as in previous studies^61,84^. At the different evaluation times, total RNA was isolated from the cultured cells and transcribed into cDNA using the NucleoSpin RNA Mini kit (Macherey–Nagel GmbH & Co., Germany) and TOPscrip RT DryMIX (Enzynomics, South Korea), respectively. Quantitative real-time PCR was carried out using TOPreal qPCR 2 × PreMIX (Enzynomics, South Korea) on a StepOnePlus real-time PCR system (Thermo Fisher Scientific) according to the manufacturer’s instructions. The relative expression was calculated by the comparative Ct (2 − ΔΔCt) method with glyceraldehyde 3-phosphate dehydrogenase (GAPDH) as the internal control. The primer sequences used are listed in Table 1.

**Table 1:** List of primer sequences used for in vitro and in vivo osteogenic gene expression analysis.

Primer	Primer sequences	
	Forward	Reverse
Human collagen-I	5′-CAG CCG CTT CAC CTA CAG C-3′	5′-TTT TGT ATT CAA TCA CTG TCT TGC C-3′
Human osteocalcin	5′-ACA CTC CTC GCC CTA TTG-3′	5′-GAT GTG GTC AGC CAA CTC-3′
Human osteopontin	5′-CTC AGG CCA GTT GCA GCC-3′	5′-CAA AAG CAA ATC ACT GCA ATT CTC-3′
Human GAPDH	5′-ACA GTC AGC CGC ATC TTC TT-3′	5′-GAC AAG CTT CCC GTT CTC AG-3′
Rabbit osteopontin	5′-GCTCGATGGCTAGCTTGTCT-3′	5′-ACAATATAAGCGCGAGGCCA-3′
Rabbit osteocalcin	5′-GTTCCCTTCCTCCTTGATTT-3′	5′-TCTACCAGTTGCAGCCTGAC-3′
Rabbit beta-actin	5′-CAGGAAGGAGGGCTGGAACA-3′	5′-ATCGTGCGGGACATCAAGGA-3′

### In vivo critical-sized bone defect model

The critical-sized bone defect model was carried out in rabbit femoral condyles for assessing the ability of g-C3N4 and GO to stimulate bone regeneration. Animal experiments were approved by the Institutional Animal Care and Use Committee of Research Facilities, Faculty of Veterinary Medicine, Assiut University, Egypt according to the Egyptian bylaws, OIE standards, and the Animal Research: Reporting of In Vivo Experiments (ARRIVE) guidelines for use of animals in research (Approval number: 06/2023/0025). All methods were performed in accordance with the relevant guidelines and regulations. In this experiment, 54 male New Zealand white rabbits (6 months, 2.5–3.0 kg) were used. They were housed in single stainless-steel cages in a well-ventilated room and maintained on a standard commercial rabbit chow diet and access to water was ad libitum. The animals’ hindlimbs were examined radiographically prior to surgery to ensure the skeletal maturity of the animals and normal bone anatomy. Rabbits were acclimatized for 2 weeks before the surgical procedure at their new housing units. Animals were randomly classified into three groups (n = 18 for each group); the control group, the g-C_3_N_4_ group, and the GO group.

Rabbits were subjected to food starvation for 8 h before surgery with free access to water. All surgical procedures were conducted under general anesthesia and strict aseptic conditions. Animals were anesthetized using a combination of xylazine HCl (3 mg/kg, Xyla-ject 20%: ADWIA Co., Egypt) and ketamine HCl (40 mg/kg, Ketamine 50%: Sigma-Tec, Egypt), and maintained using isoflurane (2.5–3%, Forane: AbbVie, England) in oxygen (2 L/minute) throughout the surgery. Following induction of general anesthesia, the right hindlimb was prepared aseptically and draped. A critical-sized bone defect model of 5 mm in diameter and 10 mm in depth was induced in right lateral femoral condyles^62^. Briefly, a lateral parapatellar 5 cm skin incision was performed on each lateral femoral condyle, then the underlying fascia was dissected parallel to the skin incision and the femoral shaft. The femoral condyle was consequently exposed by extending the muscle fibers, and the overlaying periosteum was removed. A unicortical cylindrical defect was created using a trephine burr in the center of each femoral condyle. Drilling was done using a dental micromotor with low-speed contra (Strong, Korea) and trephine burrs with continuous physiological saline irrigation to minimize thermal damage and prevent bone necrosis. Firstly, a confined cancellous defect was stepwise drilled with a trephine burr (Ø = 3 mm; Osung, Korea) perpendicular to the long axis of the femoral shaft. Then, the defects were expanded with a larger trephine burr (Ø = 5 mm; Oxy, Italy), eventually creating a critical-sized defect (Ø 5 × 10 mm). The bleeding was controlled by applying firm pressure with sterile gauze for 3–5 min. The defect was thoroughly irrigated with normal saline and dried with sterile gauze to remove debris. Then, the defects were left empty as a control group or implanted randomly with the different scaffolds (g-C_3_N_4_ or GO). At last, muscle attachment, subcutaneous tissue, and skin were routinely sutured in layers. After surgery, subcutaneous administration of meloxicam (0.6 mg/kg; Mobitil, MUP, Egypt) and penicillin (40 mg/kg; Pen & Strep, Norbook, Egypt) was carried out for five consecutive days to relieve pain and prevent infection, respectively. Animals were allowed to move inside their cages without restriction and given their traditional regimen of food and water a few hours after the operation. Rabbits were sacrificed after 4, 8, and 12 weeks of surgery (n = 6 rabbits for each time point in each group), and samples were harvested for evaluation of bone repair.

### Clinical observation

All animals were subjected to the daily clinical examination of any surgical complications, including evidence of infection and wound dehiscence. Additionally, the health condition of animals was recorded, including activity level, gait, and mobility of the hindlimbs.

### Radiographical assessment

Rabbit femoral condyles were radiographed to evaluate new bone formation in bone defects based on radiopacity changes in central and peripheral areas of the defects. Latero-medial (LM) radiographs were obtained immediately after surgery (baseline) as well as at week 4, week 8, and week 12 postoperatively. LM radiographs were taken using an ultra-high-definition film and fixed X-ray apparatus (Philips Super 80 CP, Germany). Radiographs were compared for bone density based on the mean grayscale value per unit area of defects using ImageJ 1.52 software (National Institute of Health, USA, n = 3 for each group at each time point) as reported by Liu et al.^85^.

### Computed tomography (CT) scanning

Femoral condyle bone defects were radiographed using CT scanning at week 12 using CT apparatus (120 kV and 53 mA/s, 1 mm thickness; Philips 128 slice scanner, Germany). Coronal, sagittal, and transverse images were obtained as well as lateral 3D-CT images to evaluate the bone regeneration at the defect regions.

### Gross examination of the bone defects

After sacrificing animals at different time points, the femurs were resected. All tissues were stripped for gross examination of the femoral condyles to evaluate scaffold incorporation and new bone formation.

### Histological examination

Femoral condyle samples (n = 3 for each time point) were collected and fixed in 10% neutral buffered formalin. The samples were then decalcified in 25% formic acid for 30 days at 37 °C and a pH of 7.0, dehydrated in ascending grades of ethanol, cleared in methyl benzoate, embedded in paraffin, and sectioned at 5 μm in thickness. The slides were then stained with hematoxylin and eosin (H&E) to analyze the bone formation, the response of the graft materials, and the local tissue reaction through observation of the cellular components, neovascularization, and general structure in the defect site^86^. Moreover, histochemical staining of bone collagen was performed using Crossmon's trichrome staining to examine the bone collagen fibers formation within the defect area^86^, and Sirus red staining to differentiate between mature and immature bone collagen^87^. Additionally, Periodic acid-Schiff (PAS) staining was used for demonstration of neutral mucopolysaccharide^88^. Afterward, the slides were examined under the microscope (Olympus BX51, Japan) and photographed using a digital camera (Olympus DP72, Japan). The histological interpretation was performed blindly on coded samples and compared with the control group.

### Histomorphometric analysis

The percentage of osteoid tissue area (Os%) and residual material area (RM%) were measured in the H&E-stained slides (n = 3), whereas the percentage of newly formed mature bone collagen area (Col%) was calculated in Sirius red-stained sections (n = 3). The area of osteoid tissue, the area of newly formed mature bone collagen, and the area of residual material were outlined using threshold area fraction in a specified region of interest (ROI, the entire defect area: a rectangular of 25.6 mm^2^) using ImageJ software^61,62,89^. The Os%, Col%, and RM% were reported as a percentage of the entire defect area, expressed as mean ± SD, and calculated as follows:

$${\text{Os }}\% \, = {\text{ Os}}/{\text{ROI }} \times { 1}00,{\text{ Col }}\% \, = {\text{ Col}}/{\text{ROI }} \times { 1}00,{\text{ RM }}\% \, = {\text{ RM}}/{\text{ROI }} \times { 1}00,$$

where the osteoid tissue area, newly formed mature collagen area, and residual material area were represented by Os, Col, and RM, respectively.

### Immunohistochemical evaluation (IHC)

IHC detection of CD34 in paraffin sections was performed at week 12 as described previously^90^ using CD34 monoclonal antibody (Catalog Number: CM 084 A, B, C, Biocare Medical, USA) and ultravision one detection system HRP polymer and AEC chromogen (Catalog Number: TL-015-HAJ, Thermo Fisher Scientific, USA).

### Quantitative real-time PCR (qRT-PCR) analysis

Three samples were harvested from each group on weeks 4, 8, and 12 after surgery for qRT-PCR analysis. After dissection of soft tissues and periosteum, samples were removed carefully using a trephine under strict aseptic conditions and then immediately submerged in RNA later solution (Thermo Scientific, USA) and frozen at − 80 °C for later processing. The stored frozen samples were ground under liquid nitrogen using mortar for RNA isolation. Total RNA was isolated from the samples using the TRIzol Reagent (Life Technologies) according to the manufacturer's protocol. Then, reverse transcription and qRT-PCR were performed as previously mentioned for OC and OP. The primer sequences used are listed in Table 1.

### Statistical analysis

Data were analyzed with a statistical software (IBM SPSS version 21). The data are presented as a mean ± standard deviation (SD) at a significant level of p < 0.05. The results of cytotoxicity assay (n = 8), hemocompatibility, and in vitro Col-I, OC, and OP expressions (n = 3 for each time point in each group), were compared by one-way ANOVA, followed by Tukey’s test. The results of bone density, Os%, Col%, RM%, and in vivo OC and OP expressions (n = 3 for each time point in each group) were analyzed by two-way ANOVA, followed by Tukey’s test.

## Supplementary Information

Supplementary Figures.

## Data Availability

The data that support the findings of this study are available from the corresponding author upon reasonable request.
