# Supplementary material for: Repair of critical-sized bone defects in rabbit femurs using graphitic carbon nitride (g-C3N4) and graphene oxide (GO) nanomaterials
Source: Sci Rep. 2023 Apr 3;13:5404. doi: 10.1038/s41598-023-32487-7 (PMC10070441; doi:10.1038/s41598-023-32487-7)
Supplement: Supplementary file 1 — Supplementary Figures. [file 41598_2023_32487_MOESM1_ESM.pdf]

Supplementary Figures

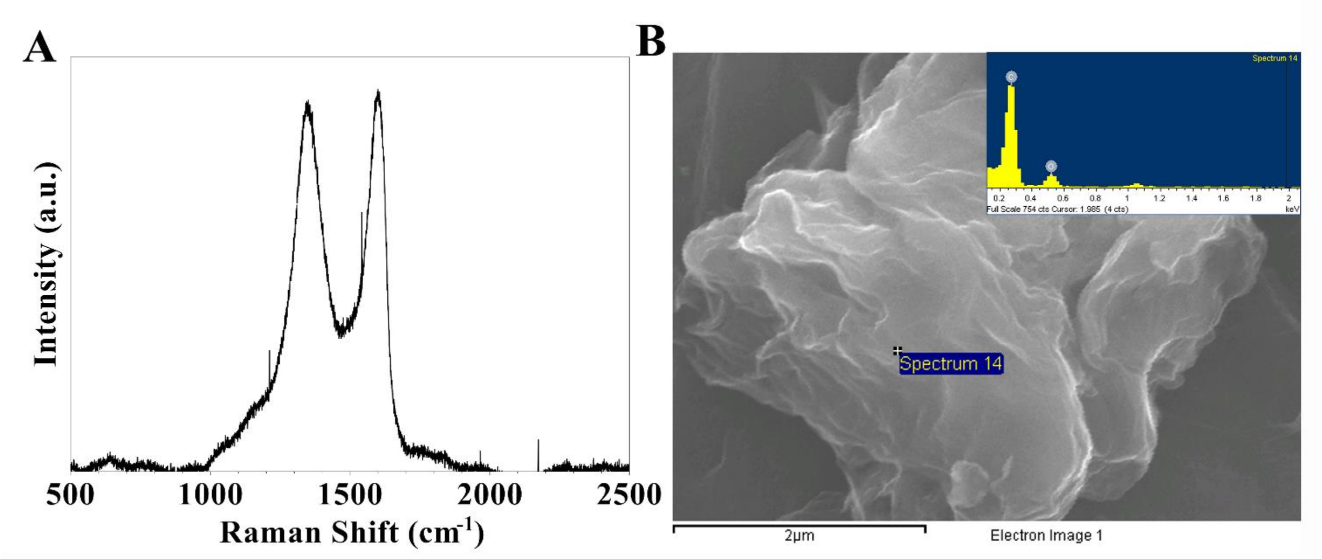

Fig. supp 1. A) Raman spectrum and B) SEM image and EDX analysis for GO.

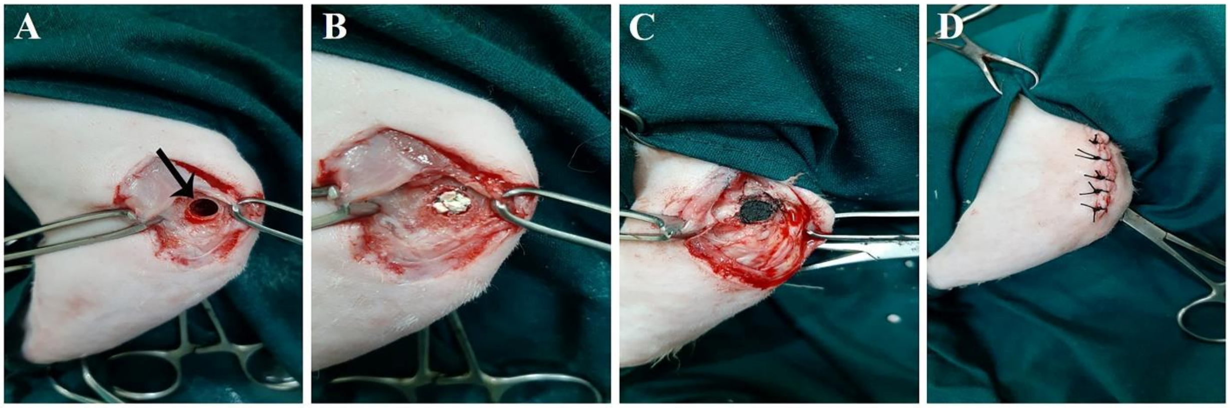

Fig. supp 2. Surgical procedure of femoral condyle bone defect induction and implantation of scaffolds; bone defect (Ø 5 × 10 mm) (black arrow) (A), g-C3N4 implantation (B), GO implantation (C), and wound closure (D).

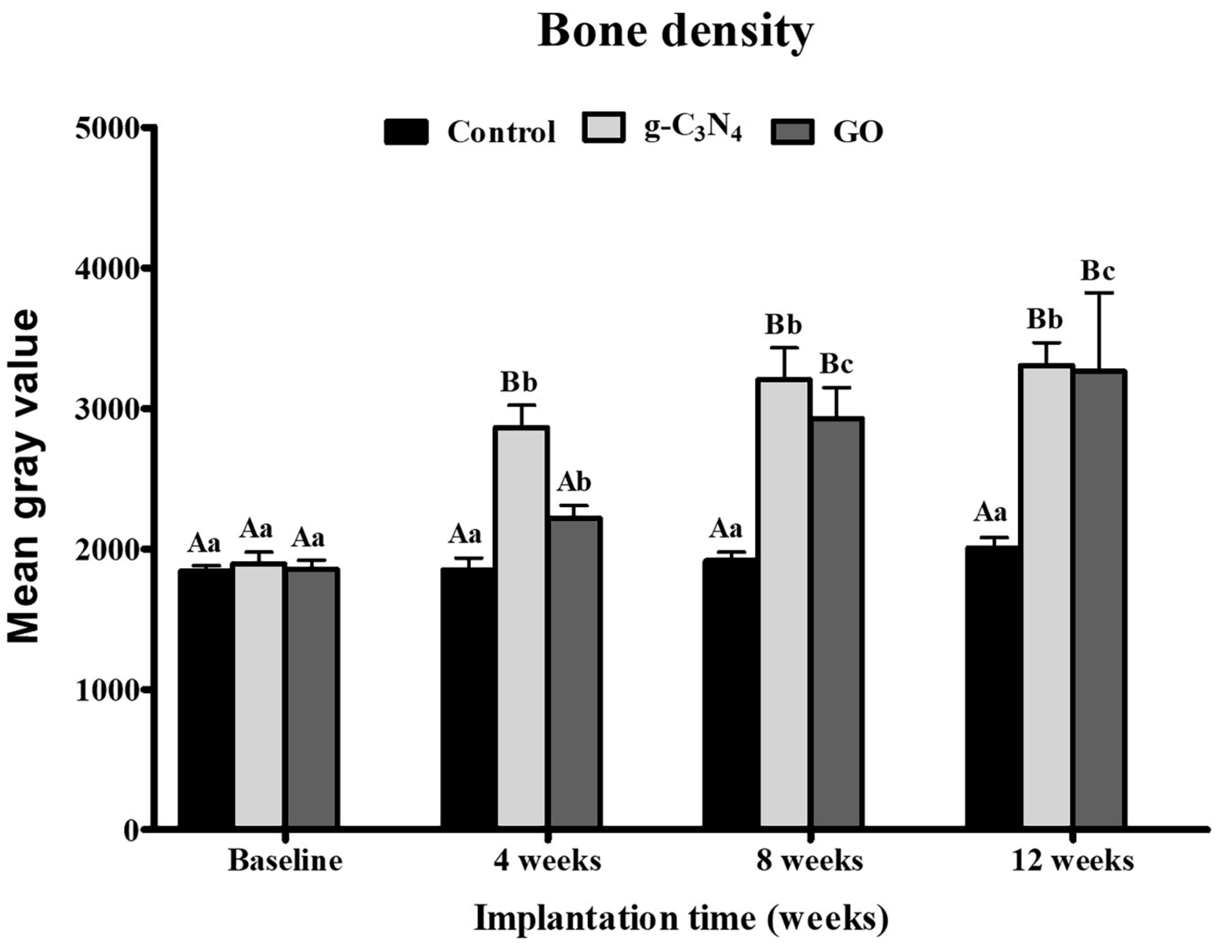

Fig. supp 3. Bone density using the mean gray value in different groups at week 4, 8, and 12 after the operation. Error bars ± SD; n = 3 for each group and time point. Bars with the same letter represent values that are not significantly different (two-way ANOVA followed by Tukey's HSD post hoc test). A, B, and C: significance between groups; a, b, and c: significance between time points.

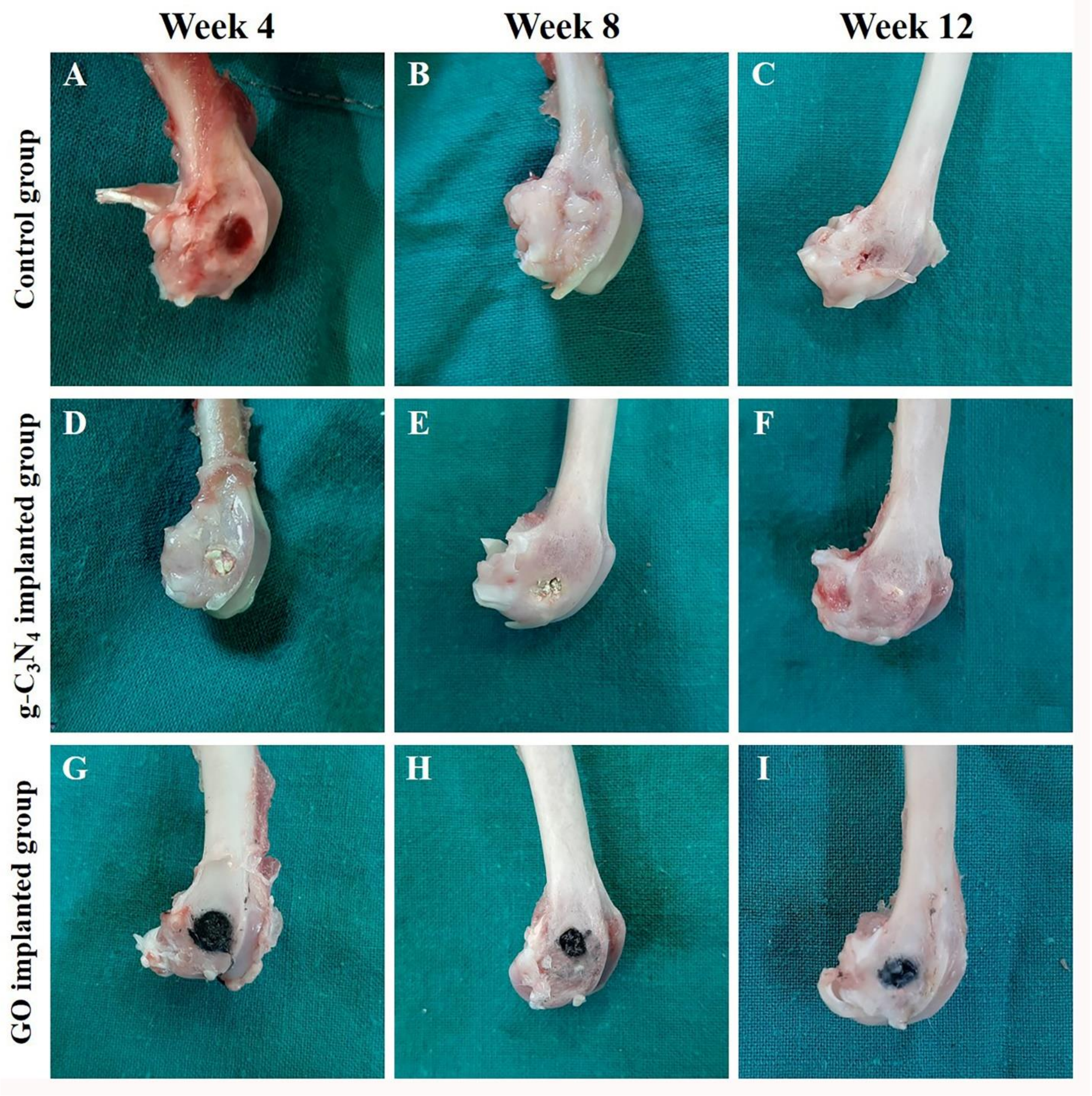

Fig. supp 4. Gross evaluation of the rabbit femoral bone defects of control (A-C), g-C_3N_4 implanted (D-F), and GO (G-I) implanted groups at different implantation times.

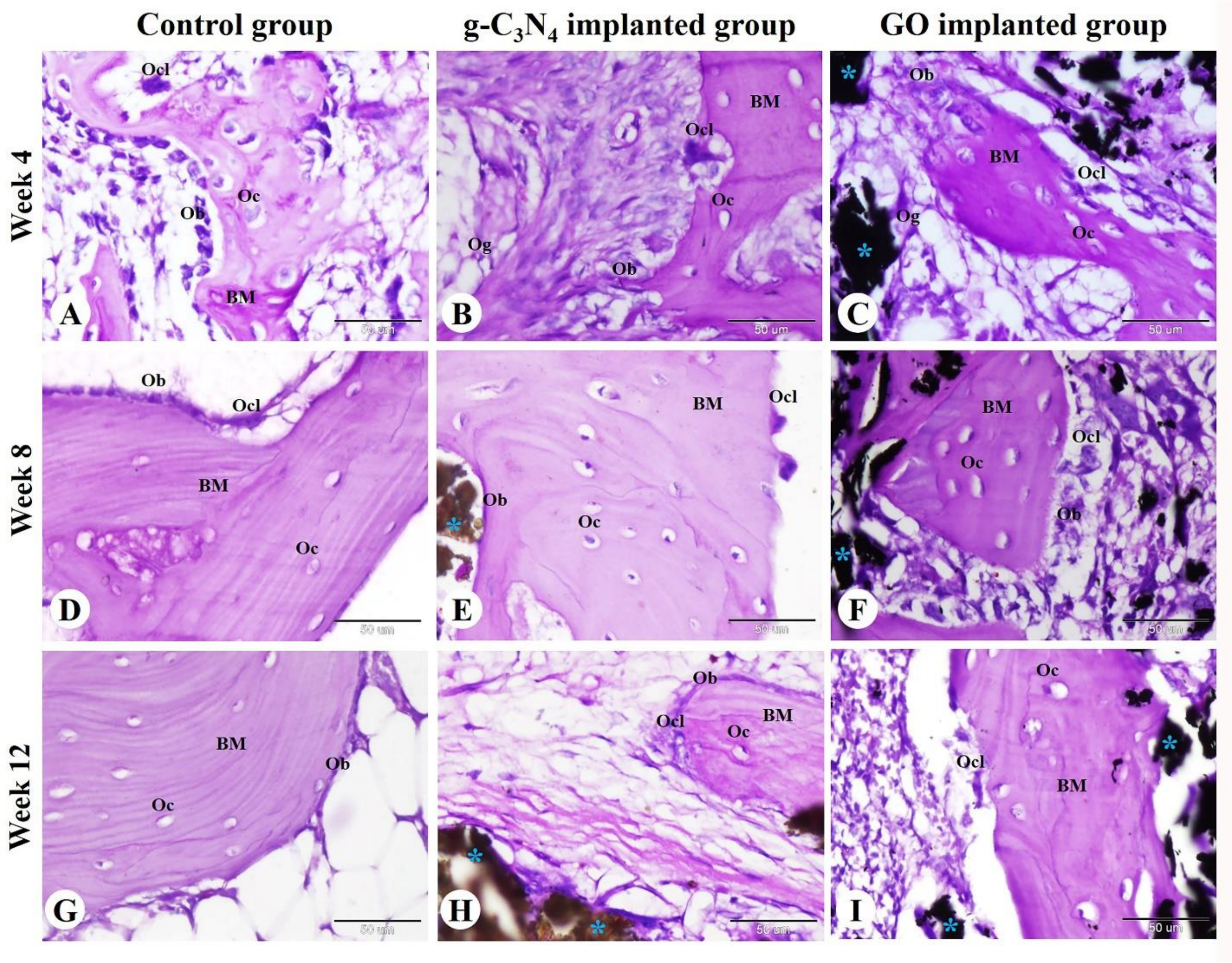

Fig. supp 5. Histological evaluation of rabbit femoral condyle defect stained with PAS & Hematoxylin. The repair site of the femoral condyle at week 4 (A-C), 8 (D-F), and 12 (G-I) after surgery in control, g-C_3N_4 implanted, and GO implanted groups. Ob: osteoblast; Oc: osteocyte; Ocl: osteoclast; Og: osteogenic cells; BM: bone matrix; blue asterisks: implanted nanomaterial. The scale bars = 50 μm.

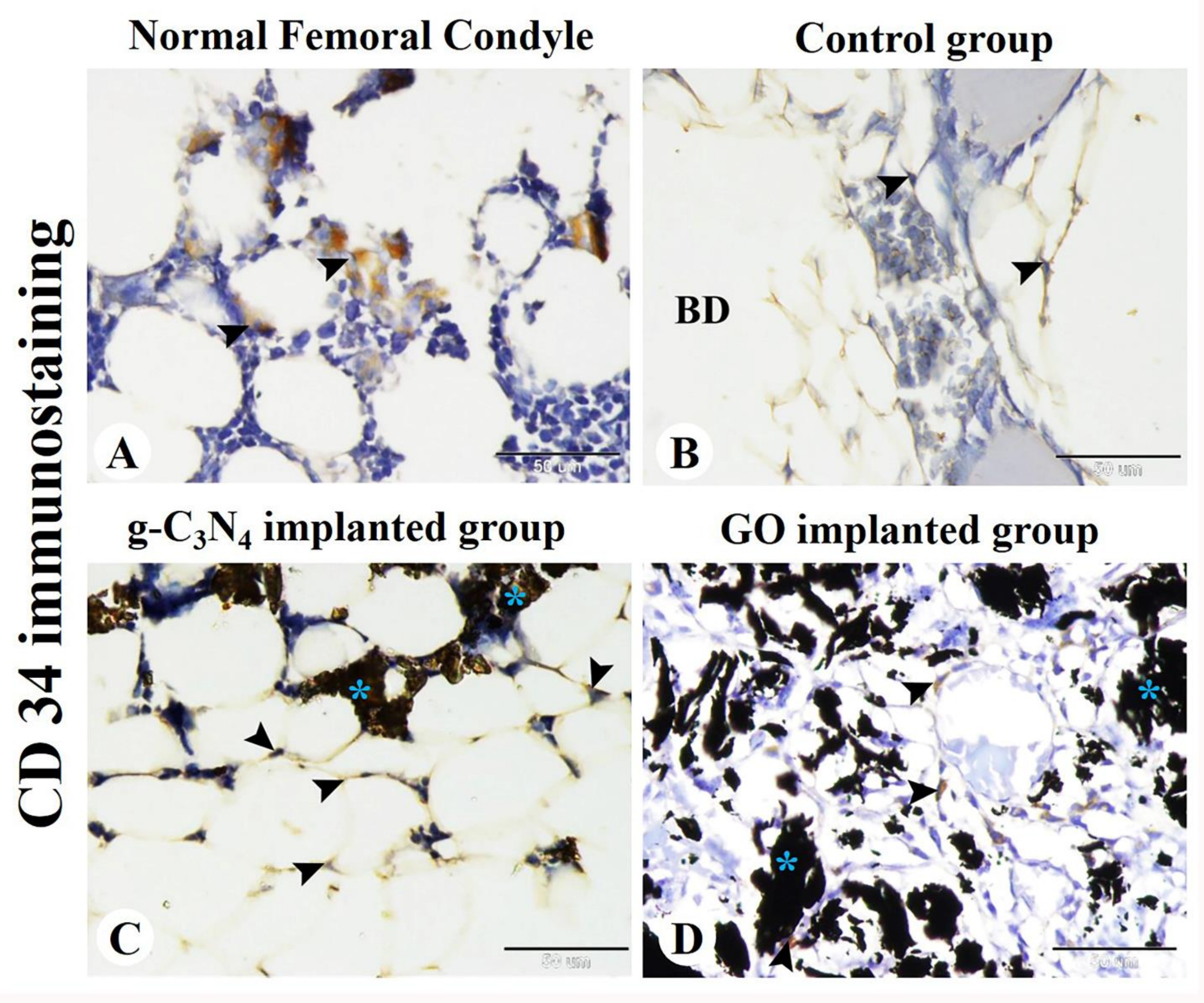

Fig. supp 6. Immunohistochemical staining of rabbit femoral condyle defect at week 12 postoperatively. The repair site of the femoral condyle in the control (B), g-C_3N_4 implanted (C), and GO implanted (C) groups was stained with CD34 monoclonal antibody. BD: bone defect; Black arrowheads: $CD34^+$ mesenchymal stem cells; blue asterisks: implanted nanomaterial. Scale bars = 50 µm.
